# Emerging roles and competencies of district and sub-district pharmacists: a case study from Cape Town

**DOI:** 10.1186/s12960-015-0081-8

**Published:** 2015-11-24

**Authors:** Hazel Bradley, Uta Lehmann, Nadine Butler

**Affiliations:** School of Public Health, University of the Western Cape, Private Bag X17, Bellville, 7535 South Africa

**Keywords:** District pharmacist, Primary health care, Roles, Competencies, Participatory action research, South Africa, Developing country

## Abstract

**Background:**

District and sub-district pharmacist positions were created during health sector reform in South Africa. High prevalence of HIV/AIDS, tuberculosis and increasing chronic non-communicable diseases have drawn attention to their pivotal roles in improving accessibility and appropriate use of medicines at the primary level. This research describes new roles and related competencies of district and sub-district pharmacists in Cape Town.

**Methods:**

Between 2008 and 2011, the author (HB) conducted participatory action research in Cape Town Metro District, an urban district in the Western Cape Province of South Africa, partnering with pharmacists and managers of the two government primary health care (PHC) providers. The two providers function independently delivering complementary PHC services across the entire geographic area, with one provider employing district pharmacists and the other sub-district pharmacists. After an initiation phase, the research evolved into a series of iterative cycles of action and reflection, each providing increasing understanding of district and sub-district pharmacists’ roles and competencies. Data was generated through workshops, semi-structured interviews and focus groups with pharmacists and managers which were recorded and transcribed. Thematic analysis was carried out iteratively during the 4-year engagement and triangulated with document reviews and published literature.

**Results:**

Five main roles for district and sub-district pharmacists were identified: district/sub-district management; planning, co-ordination and monitoring of pharmaceuticals; information and advice; quality assurance and clinical governance; and research (district pharmacists)/dispensing at clinics (sub-district pharmacists). Although the roles looked similar, there were important differences, reflecting the differing governance and leadership models and services of each provider. Five competency clusters were identified: professional pharmacy practice; health system and public health; management; leadership; and personal, interpersonal and cognitive competencies. Whilst professional pharmacy competencies were important, generic management and leadership competencies were considered critical for pharmacists working in these positions.

**Conclusions:**

Similar roles and competencies for district and sub-district pharmacists were identified in the two PHC providers in Cape Town, although contextual factors influenced precise specifications. These insights are important for pharmacists and managers from other districts and sub-districts in South Africa and inform health workforce planning and capacity development initiatives in countries with similar health systems.

## Background

District and sub-district pharmacist positions have been created across South Africa since 1994 as part of health sector reform in which health services have moved from a fragmented, hospital-based service to a primary health care (PHC) approach based on the district health system [1]. The country is divided into 53 health districts, each comprising a number of sub-districts, and district management teams (DMTs) established with the responsibility for strategic and operational functions in the district [2]. District health services include community-based services, primary health care services – delivered from clinics and community health centres and district hospitals. Within the DMT, district and sub-district pharmacists generally have responsibility for medicine supply and management at the PHC level, with other pharmacists, pharmacy support workers and nurses performing a variety of tasks such as ordering of medicines, stock management and dispensing [3]. These government district health structures provide for the health needs of approximately 85% of South Africa’s population, with PHC services free at the point of care [4]. The remaining 15% of the population have private health insurance and utilize private general practitioners, retail pharmacies and private hospitals.

From the mid-1990s onwards, district pharmacists have been appointed as members of DMTs in several sub-Saharan African countries, including Tanzania, Ghana, Kenya, Uganda, Malawi and Zimbabwe as health reforms were implemented [5–7]. Key roles included membership of the district management team, medicine supply, rational medicine use and training and supervision of medicine-related activities at the PHC level. The increase of pharmacy support workers and community health workers involved in medicine supply and distribution, as a result of task sharing and task shifting to mitigate shortages of pharmacists and improve access to essential medicines, reinforces the importance of the supervisory role [8]. In Tanzania, district pharmacists were involved in planning, budgeting, management and support of health services in the district [9]. However, in most countries, there is little specific information about district pharmacists’ responsibilities and how they function within the management team. This is despite a number of World Health Organization (WHO)-led Consultative Workshops on The Role of the Pharmacist which advocated for the importance of pharmacists in developing countries having population-level responsibilities [10].

In South Africa, several organizations and academic institutions have contributed to the establishment of district pharmacists in the country since 1994 [11]. Health Systems Trust (HST) worked with several districts to provide practical guidance and tools for implementing drug management within districts [12, 13]. Significantly, they advocated that each district identify one person responsible for medicines, initially called a district drugs controller, and that this person should be a member of the district management team [13]. Their key roles included the following: monitoring and evaluating drug supply and distribution, developing and implementing standard procedures for drug management, identifying training needs, developing delivery schedules, liaising with medical officers and nurse practitioners and reporting to the DMT on drug management. Unfortunately, these early initiatives were not taken forward consistently across the country, or sustained over time, resulting in the nine provinces appointing district and sub-district pharmacists at different occupational (and salary) levels, with continuing uncertainty about their responsibilities [14].

Considering this evolution of district pharmacists’ roles in the region over the past 20 years, it is surprising that little information is available about the competencies that pharmacists require to function optimally in these positions or initiatives to develop competencies. However, globally, numerous competency frameworks for pharmacists working in a variety of settings have been developed. As the roles of district and sub-district pharmacists were somewhat comparable to those identified for primary care pharmacists working in the United Kingdom (UK), the competency framework developed for these cadres in 2000 was interrogated [15]. The Advanced and Consultant Level Framework adapted for pharmacists in hospital and primary care management, also from the UK, was relevant to this research as district and sub-district pharmacists are generally experienced practitioners working in primary health care management positions [16]. The latter framework comprises six competency clusters: expert professional management; building working relationships; leadership; management; education, training and development; and research and evaluation. In addition, several management competency frameworks were identified including a framework for district mangers in South Africa which was published towards end of the research. It included three main competency clusters: core, management and leadership [17]. These competency frameworks, in particular, were used to inform the research.

The pivotal roles of district and sub-district pharmacists in South Africa have been highlighted by the increasing burden of chronic diseases, human immunodeficiency virus/acquired immune deficiency syndrome (HIV/AIDS) and non-communicable diseases requiring regular medication [18]. In addition, recent national initiatives focusing on revitalizing PHC, and National Health Insurance, a form of universal health coverage, have renewed focus on health districts and the impetus to strengthen DMTs across the country [19, 20]. However, despite recognition during the late 1990s that new cadres, including pharmacists, working at district and sub-district levels would require the re-definition of roles and functions, the progress with the establishment of new cadres capable of performing the required services has been limited [21, 22]. This paper describes the systematic identification of new roles and related competencies of district and sub-district pharmacists in Cape Town, during a period of structural re-organization. It outlines the participatory action research approach used in this study – two subsequent papers will discuss the application of this methodology to inform pharmacy practice in a changing health system and implications for pharmacists moving into these new positions.

## Methods

### Research setting

The research was set in Cape Town, a metropolitan district with a population of 3.8 million, in the Western Cape Province of South Africa. Two tiers of government, the local authority, City Health, and the Western Cape provincial department of health, Metro District Health Services (MDHS), provide complementary PHC services, free at the point of care across the same geographic area, to approximately 85% of the population. The remaining population accesses private health care providers including general practitioners, retail pharmacies and private hospitals [4]. The two PHC providers currently function independently across the whole district, although there are good working relationships at management and operational levels. The research took place in the context of ongoing district development in Cape Town as the two PHC providers were at different stages of establishing district and sub-district pharmacists. City Health appointed four sub-district pharmacists in 2005, prior to the commencement of this research, each responsible for two sub-districts. In 2009, midway into the research, MDHS was divided into four sub-structures (each comprising two sub-districts) and a sub-structure pharmacist^1^ was appointed into each of the four management teams (Table 1).

**Table 1:** **Description Cape Town PHC providers and types of services offered**

**Provider**	**Type of service**	**Hospitals**	**Community health centres**	**Clinics**	**Pharmacists**
City Health	Preventive	0	3	100	4^a^
Metro District Health Services	Curative	9	42	0	4^b^

^a^Sub-district pharmacists.

^b^District pharmacists.

### Research approach

Between 2008 and 2011, the author (HB) conducted participatory action research (PAR), partnering with pharmacists and managers of both government PHC providers in Cape Town as well as representatives from the provincial level and non-governmental organizations (NGOs) [23]. A key feature of PAR is collaborative learning through iterative cycles of action and reflection in which change is generated through new learning and empowering participants [24]. This approach acknowledges the importance of context and dynamic interplay of elements of the health system and was thus suited to this case study in which the roles and related competencies of district and sub-district pharmacists were identified as they unfolded in the two PHC organizations [25, 26].

### Data collection and analysis

After an initiation stage, the research evolved into a series of five iterative cycles of action and reflection, each providing increasing understanding of the roles and related competencies of district and sub-district pharmacists and their experiences as they transitioned into these new management positions in the two organizations (Table 2) [27].

**Table 2:** **Overview of research project cycles**

**Initiating the project**	
Cycle 1	Identifying roles of district & sub-district pharmacists
Cycle 2	Identifying competencies of district & sub-district pharmacists
Cycle 3	1^st^ reflection on roles and competencies of district & sub-district pharmacists
Cycle 4	Developing & piloting intervention to enhance competencies
Cycle 5	2^nd^ reflection on roles and competencies of district & sub-district pharmacists

The research centred around two series of three interactive workshops, with each workshop lasting about 3 h and attended by between 9 and 17 pharmacists and managers from both PHC providers. In accordance with methodological approach proposed by Whiddett and Hollyforde, participants first identified the roles of the two cadres and then used these to identify competencies required to fulfil these roles [28]. Thirty-nine semi-structured interviews with pharmacists and managers from both PHC providers and two focus groups, one each with district pharmacists (four participants) and sub-district pharmacists (four participants), were conducted. All semi-structured interviews were recorded and transcribed verbatim; focus groups were recorded and the recordings used to enhance notes taken during the focus groups. The purpose of the interviews and focus groups was to inform conceptualization, supplement workshops and reflect on experiences. In addition, all official health policy and planning documents and reports on governance, health systems and human resources for health and medicines published by the South African government, the provincial government of the Western Cape and the South Africa Pharmacy Council post-1994 were reviewed to identify aspects likely to influence pharmacists’ roles and required competencies at the PHC level.

Thematic analysis of the interviews and focus groups was carried out iteratively and contributed to the workshops along with information from the documentary review and literature search [29]. In this way, all information was brought into the participatory process providing opportunities for collaborative analysis and interpretation. The integration of literature into data analysis and interpretation is a characteristic of action research – called “dialectical analysis” by Winter [30]. This use of literature during analysis and interpretation strengthens the confidence of the final outcomes [31].

Techniques applied to ensure quality of research in this study included transparency (a clear account of study procedures and audit trail), member checking (feeding back findings to participants for verification and to inform decisions of the next stage), triangulation (use of different research methods and sources) and critical self-reflexivity [32–34]. Critical self-reflexivity, where perspectives of the researcher are fully acknowledged throughout the research, was particularly important in this study and the lead researcher’s (HB) background as a pharmacist and previous with experience of pharmaceutical services in the UK and South Africa were acknowledged during the research [35]. In addition, whilst this methodological approach does not support generalizability, Lincoln and Guba maintain that the findings are transferable to similar settings [36].

### Ethical considerations

The research was approved by the University of the Western Cape Research and Ethics Committees (Registration No. 8/1/10) and permission granted from MDHS and City Health. All participants gave their written consent to participate after the ethical issues of the emergent processes of action research had been discussed.

## Results and discussion

### Roles of district and sub-district pharmacists

Five main roles for district and sub-district pharmacists emerged (Table 3). Four of these were similar for district and sub-district pharmacists: contribution to district and sub-district management team; planning, co-ordination and monitoring of pharmaceuticals; provision of information and advice; and participation in quality assurance and clinical governance. The fifth role was different and for district pharmacists; this was participation in research, and for sub-district pharmacists, it was dispensing medicines at the clinics.

**Table 3:** **Roles of district and sub-district pharmacists**

**District pharmacists (MDHS)**	**Sub-district pharmacists (City Health)**
*Contribute to district management team*	*Contribute to sub-district management team*
*Planning, co-ordination and monitoring of:*	*Planning, management and monitoring of:*
Pharmaceuticals	Pharmaceuticals
Pharmaceutical human resources	Pharmaceutical human resources
Pharmaceutical budget	Pharmaceutical budget
Pharmaceutical infrastructure	Pharmaceutical infrastructure
*Provide information and advice on professional, legal, clinical and technical aspects to:*	*Provide information and advice on professional, legal, clinical and technical aspects to:*
Health managers	Health managers
Health workers	Health workers
Health programmes	Health programmes
NGOs	NGOs
Private providers	Private providers
Consumers	Consumers
*Participate in quality assurance and clinical governance*	*Participate in quality assurance and clinical governance*
*Participate in research activities*	*Dispense medicines at clinics*

Whilst these broad categories of roles for district and sub-district pharmacists ostensibly looked similar, and were not very different from those proposed for district drug co-ordinators and district pharmacists from South Africa and several sub-Saharan African countries, there were substantial differences between the district and sub-district pharmacists’ roles in the two organizations [13, 14]. District pharmacists were generally more involved in strategic-level management functions, whilst sub-district pharmacists combined sub-district management activities with some “hands-on” dispensing at clinics. Essentially, the two cadres were working at different management and leadership levels, with district pharmacists working at the middle management level and sub-district pharmacists straddling first and middle management levels [37].

The difference in roles appeared to be shaped by several contextual factors including the nature and organization of the PHC services as well as differences in governance and leadership in the two providers. MDHS, for example, provides mainly curative services from its facilities. A major focus at community health centres is the management of adults with chronic non-communicable diseases, with each having its own medical staff and registered pharmacy staffed by pharmacists and pharmacist’s assistants. District pharmacists were not expected to work at facilities but to play an active role in the DMT which was facilitated by their offices being located at the district office.

In City Health, on the other hand, clinics provide nurse-driven promotive and preventive services, such as child immunizations, reproductive and sexual health services and tuberculosis treatment. Most clinics have part-time medical cover and do not have registered pharmacies or pharmacists. Vaccines and medicines are usually managed by a professional nurse, with increasing numbers of pharmacists’ assistants being assigned to clinics. This set-up consequently requires the sub-district pharmacists to play a greater supervisory role with regular visits to the clinics to provide hands-on support than is the case in MDHS. In some sub-districts, there was an expectation that sub-district pharmacists provide some dispensing services at clinics, although this was acknowledged by most managers and sub-district pharmacists as problematic. Juggling mid-level managerial responsibilities and clinical service provision has been highlighted as challenging in other settings in sub-Saharan Africa. In addition, in City Health, most sub-district pharmacists were based at clinics, rather than the sub-district office, and had restricted access to transport. This practical arrangement also limited sub-district pharmacists’ ability to fully engage with sub-district management activities compared to the district pharmacists in the other organization who were based at the district offices.

Overall, these new management roles required pharmacists to shift from direct service provision to move towards management and support functions, and from interacting within the pharmaceutical profession to working as part of a multi-professional team to oversee delivery of pharmaceutical services across their district or sub-district. One pharmacist articulated his new role as follows: “If there is a pharmacy that renders a service we need to support it…build capacity at CHC [community health centre] level so they can function as a team” (M2P:175). However, the same pharmacist was pragmatic in his approach by saying: “So, depending on the strength and capacity of the facility manager, some of it spills over into my domain and then you need to do something because, otherwise you know the pharmacy is not working” (M2P:175).

Significantly, district and sub-district pharmacists’ roles in their organizations evolved over time. One manager in MDHS, speaking on behalf of his colleagues, highlighted this emergent process, two years after district pharmacists had been appointed, by saying: “I think most managers will tell you the same thing. I think there’s an improvement, in first of all their [pharmacists] understanding of their role…they understand that they are middle managers and if you are a middle manager there are clearly defined functions and responsibilities …” (M1M:176). Another manager affirmed the establishment of his district pharmacist’s position by saying: “…what we are seeing now in practice is that our pharmacist is a fully-fledged member of the district management team” (M3M:173).

### Competencies of district and sub-district pharmacists

The second stage of the research identified competencies that district and sub-district pharmacists required to fulfill their new roles. As largely similar roles for district and sub-district pharmacists were identified, it was not surprising that five comparable competency clusters were identified but with different emphases between the two cadres (Table 4).

**Table 4:** **Competencies of district and sub-district pharmacists**

*Professional pharmacy practice*	*Personal, interpersonal, cognitive*
Legal and regulatory aspects of pharmacy practice	*Personal*
Clinical aspects of pharmacy practice	Self-development
Technical aspects of pharmacy practice	Adaptability
Public service legislation and practice	Assertiveness
*Health system/public health*	Time management
Health systems and organization	Professionalism
Health programmes	*Interpersonal*
Public health	Relationship building
*Management*	Networking
Planning and organizing	Negotiation
Financial management and budgeting	Teamwork
Human resources management	Cultural competency
Physical resources management	*Cognitive*
Project management	Problem solving
Information management	Prioritizing
Monitoring and evaluation	Decision making
*Leadership*	Communication skills (oral and written)
Strategic leadership and vision	
Change management	
Service development and innovation	

One of the competency clusters, professional pharmacy practice, was pharmacist specific, whilst the other four were generic and could apply to other managers working at the district or sub-district level. District and sub-district managers valued the professional pharmaceutical expertise pharmacists brought to the management team, especially regarding planning and establishment of new services and facilities and participating in clinical governance and quality assurance initiatives. One manager illustrated the contribution of his pharmacist to the district team by saying: “… before, the immunization campaigns, particularly around influenza, they were not well managed and we often ended up with stock being wasted. It changed last year, and this year it changed completely, everything was used, virtually everything was used” (M3M:173). Pharmacists, on the other hand, commented that these population-focused competencies were different from those gained in pre-service training where, in line with other health professional training, the main focus was on individual-level pharmaceutical care [38].

Given the focus of district and sub-district pharmacists’ roles, it was not surprising that management and leadership competencies were identified as critical. Although there is a close relationship between management and leadership, there are differences and many involved in health system strengthening maintain that health systems undergoing change, as is the case in Cape Town, require managers with both these competencies [37, 39]. Management capacity at district levels has for some time been recognized as key to delivering quality health services, both in South Africa and globally [40, 41]. However, over the past few years, there has been increasing acknowledgement of the need to strengthen management and leadership capacity in the South African health system with several national-led initiatives including the establishment of the Academy for Leadership and Management in Healthcare [42]. In addition, Management Sciences for Health (MSH)/Systems for Improved Access to Pharmaceuticals and Services (SIAPS) recently launched a tailored Leadership Development Programme for pharmacists to support the development of workplace management capacity to improve the quality of pharmaceutical services in several provinces in South Africa.

As the research progressed iteratively, the significance of personal, interpersonal and cognitive competencies was recognized by pharmacists and managers. Mintzberg similarly identified cognitive competencies as essential for leaders and managers, and this aligns with recent health-system-strengthening initiatives highlighting the building of systems “software”, in addition to “hardware”, as critical for organizational performance [43]. Whereas there was little emphasis in the development of these so-called “soft skills” in the past, the importance of including these skills in training health professionals, including pharmacists, is becoming more widely acknowledged, although it has still to gain formal recognition in South Africa [38, 44].

## Conclusions

This research highlighted the nuances and complexities of the roles and competencies of district and sub-district pharmacists working at the two PHC providers in Cape Town by identifying roles and related competencies that looked similar but were actually quite different. In this way, it illustrated the importance of taking contextual and other health system factors into consideration when defining precise role specifications for district and sub-district pharmacist posts and suggests a similar approach to characterizing the required competencies.

These insights have implications for district and sub-district managers and pharmacists and others involved in health workforce planning and development. In particular, the research found that pharmacists working in these positions performed wide-ranging management functions as part of the district or sub-district management team, albeit with a particular responsibility for pharmaceutical services. Consequently, district and sub-district pharmacists required generic management and leadership competencies, as well as professional pharmacy practice competencies with a population-level focus, and these are different competencies from traditional pre-service training. The research emphasizes the importance of developing appropriate initiatives to build these competencies in district and sub-district pharmacists if they are to optimally perform in these positions and contribute meaningfully to improving the accessibility and rational use of medicines in their settings.

Some of the pharmacy-specific, technical management and public health competencies may be developed through traditional formal training at academic institutions such as accredited masters and postgraduate diploma courses. The “softer” relational competencies associated with management and leadership requires more innovative approaches including on-the-job training activities such as in-service training, mentoring and coaching [45]. As a result of this work, the University of the Western Cape has established an area of specialization in Pharmaceutical Public Health in its existing Master of Public Health. In addition, the South African Pharmacy Council is establishing a new pharmacist specialist in Pharmacy Public Health and Management with a masters-level qualification which should provide many of the competencies required for these positions [46].

### Endnote

^1^Due to the size and nature of management of the sub-structures, sub-structure pharmacists are equivalent to district pharmacists in other settings and will be called district pharmacists throughout this paper.

## Abbreviations

CHC: Community health centre
DMT: District management team
HIV/AIDS: Human immunodeficiency virus/acquired immune deficiency syndrome
HST: Health Systems Trust
MDHS: Metro District Health Services
MSH: Management Sciences for Health
NGOs: Non-governmental organizations
PAR: Participatory action research
PHC: Primary health care
SIAPS: Systems for Improved Access to Pharmaceuticals and Services
UK: United Kingdom
WHO: World Health Organization
